# Optimized Protocol for Proportionate CNS Cell Retrieval as a Versatile Platform for Cellular and Molecular Phenomapping in Aging and Neurodegeneration

**DOI:** 10.3390/ijms23063000

**Published:** 2022-03-10

**Authors:** Quratul Ain, Christian W. Schmeer, Diane Wengerodt, Yvonne Hofmann, Otto W. Witte, Alexandra Kretz

**Affiliations:** 1 Hans Berger Department of Neurology, Jena University Hospital, 07747 Jena, Germany; christian.schmeer@med.uni-jena.de (C.W.S.); diane.wengerodt@med.uni-jena.de (D.W.); otto.witte@med.uni-jena.de (O.W.W.); 2 Department of Internal Medicine V, Jena University Hospital, 07747 Jena, Germany; yvonne.hofmann@med.uni-jena.de

**Keywords:** amyotrophic lateral sclerosis (ALS), CNS aging, CNS cell isolation, neurodegeneration, neuropathology, progeria, senescence

## Abstract

Efficient purification of viable neural cells from the mature CNS has been historically challenging due to the heterogeneity of the inherent cell populations. Moreover, changes in cellular interconnections, membrane lipid and cholesterol compositions, compartment-specific biophysical properties, and intercellular space constituents demand technical adjustments for cell isolation at different stages of maturation and aging. Though such obstacles are addressed and partially overcome for embryonic premature and mature CNS tissues, procedural adaptations to an aged, progeroid, and degenerative CNS environment are underrepresented. Here, we describe a practical workflow for the acquisition and phenomapping of CNS neural cells at states of health, physiological and precocious aging, and genetically provoked neurodegeneration. Following recent, unprecedented evidence of post-mitotic cellular senescence (PoMiCS), the protocol appears suitable for such de novo characterization and phenotypic opposition to classical senescence. Technically, the protocol is rapid, efficient as for cellular yield and well preserves physiological cell proportions. It is suitable for a variety of downstream applications aiming at cell type-specific interrogations, including cell culture systems, Flow-FISH, flow cytometry/FACS, senescence studies, and retrieval of omic-scale DNA, RNA, and protein profiles. We expect suitability for transfer to other CNS targets and to a broad spectrum of engineered systems addressing aging, neurodegeneration, progeria, and senescence.

## 1. Introduction

The mammalian CNS is composed of several types of projection neurons, interneurons, glial populations—including oligodendrocytes, astrocytes, and microglia—and extracellular matrix molecules. Such a heterogeneous cellular network environment is now scientifically accessible, e.g., at the organoid, slice, neurosphere, primary tissue, and cell culture level [1,2,3,4,5], thereby allowing specifications towards the degree of specimen maturation and coverage of embryonic [6,7,8], postnatal [9], and juvenile stages [10]. However, protocols dedicated to the achievement of single cell isolates [11] encompassing the retrieval of neurons [1,10] instead of glia only [12], and which originate from mature [1,10], aged [13], and particularly neurodegenerative conditions are still infrequent. Similarly underrepresented are algorithms that purify anatomically confined cell entities with specific susceptibility to degeneration and death, e.g., from model systems that pinpoint the effect of certain target mutations [8,11,14]. Recent highly specialized procedural algorithms achieving, e.g., neural stem cell isolates and their neuronal differentiation on 2D films or in 3D biopolymer scaffolds [15], or those challenging the retrieval of CNS subpopulations, e.g., via translating ribosome affinity purification (TRAP) requiring artificial chromosomes in BAC transgenic animals [16,17], are often cost- and resource-intensive or depend on specific technical equipment and thus might lack accessibility to a broad majority of work facilities. Though innovative TRAP approaches [16,17] can theoretically assay any cell type of interest at any age category, its application on aged and neurodegenerative conditions is still sparse [18,19]. Thus, there is a yet unmet need for CNS cell isolation techniques that allow for a proportionate retrieval of singularized neural cells, including neurons and entity-specific subpopulations, from aging, progeroid, and neurodegenerative CNS, thereby covering isolates both from brain and spinal cord regions.

Several lines of evidence indicate profound aging-related transformations in the molecular composition of neural plasma membranes [20], in cell-shaping organelle arrangements, and the cytoskeletal architecture affecting micro-/intermediate filaments and microtubule structures. Such multiple age-related changes, which can manifest even independently of a typical signature of senescence [21], influence the biophysical and biochemical cell and tissue properties [20,21,22] and reciprocally interfere with key parameters that are crucial for the efficiency of cell isolation techniques, such as membrane fluidity, cellular viscidity, adherence, and apoptosis resistance. Likewise, there are well-described aging-associated peculiarities in the lipid profile of CNS plasmalemma, arising from alterations in their biosynthesis, neuronal vesicle uptake, and inter- and intramembrane cholesterol trafficking, as well as the catabolism and content of glycosylated sphingolipids or gangliosides and ceramides [20,23,24]. Within the CNS cellular composite, isolation of neurons and neuronal subtypes appears particularly challenging due to their susceptibility to damage imposed by mechanical stress or chemical treatments, steps that are inevitable for cell dissemination. Such intrinsic vulnerability becomes put at further vital risk under aging and senescence conditions, when neurons gradually lose their metabolic resilience, protein and ion homeostasis, and apoptotic resistance due to a life-long accumulation of damaging events, including oxidative stress and unrepaired genotoxic injuries, failure to degrade and replace misfolded or oxidized proteins and lipids or other toxic metabolites, and the formation of stress bodies and aggregates that interfere with metabolic processes vital to cell maintenance [23]. Moreover, the prevalence of aging-associated cellular byproducts in CNS tissue, particularly of auto-fluorescent lipofuscin that arises from incomplete lysosomal digestion of oxidized proteins and lipid peroxidation, can severely interfere with the cell retrieval and foreseen downstream applications, e.g., the proper use of reporter signals, the implementation of immunofluorescent protocols or FACS analyses, or with protein and nucleic acid extractions and gene profiling approaches. Thus, cell extraction protocols from aged CNS tissue require complex validations in terms of assay suitability, efficiency, and reproducibility.

A similar scenario applies to conditions of neurodegeneration that are, in human patients, often aging-related and successfully recapitulated under laboratory conditions, e.g., in genetically engineered animal models mimicking human Alzheimer’s dementia, Parkinson’s disease, and amyotrophic lateral sclerosis (ALS) [25] or progeria syndromes [26,27]. Moreover, the recent discovery that, similar to dividing cells [21], non-replicative cell fractions including differentiated neurons can adopt senescent-like phenotypical properties under stress conditions in vivo might pose a further level of consideration when brain-derived cell isolates are to be achieved [28,29,30]. Though the contribution of post-mitotic cellular senescence (PoMiCS; [29]) to the aging process and the progression of chronic CNS pathologies is still unclear [30], the phenomenon of atypical neuronal cell cycle re-induction disclosed in many age-related neurodegenerative disorders [31] suggests a paving or paralleling itinerary towards aging and senescence [32]. Methodical algorithms that specify classical senescence [33,34] are rare, and those dissecting it phenotypically from PoMiCS [29,30] are still unestablished and might, in the context of neurons, profit from protocols that conjointly isolate replicative and non-replicative cell entities from the same CNS cellular network, as introduced here by our protocol.

Thus, considering the rising importance of aging- and senescence-related scientific topics and those centered on the pathophysiology of neurodegenerative disorders, adaptations to respective cell isolation protocols appear to be indispensable.

**Figure 1:**
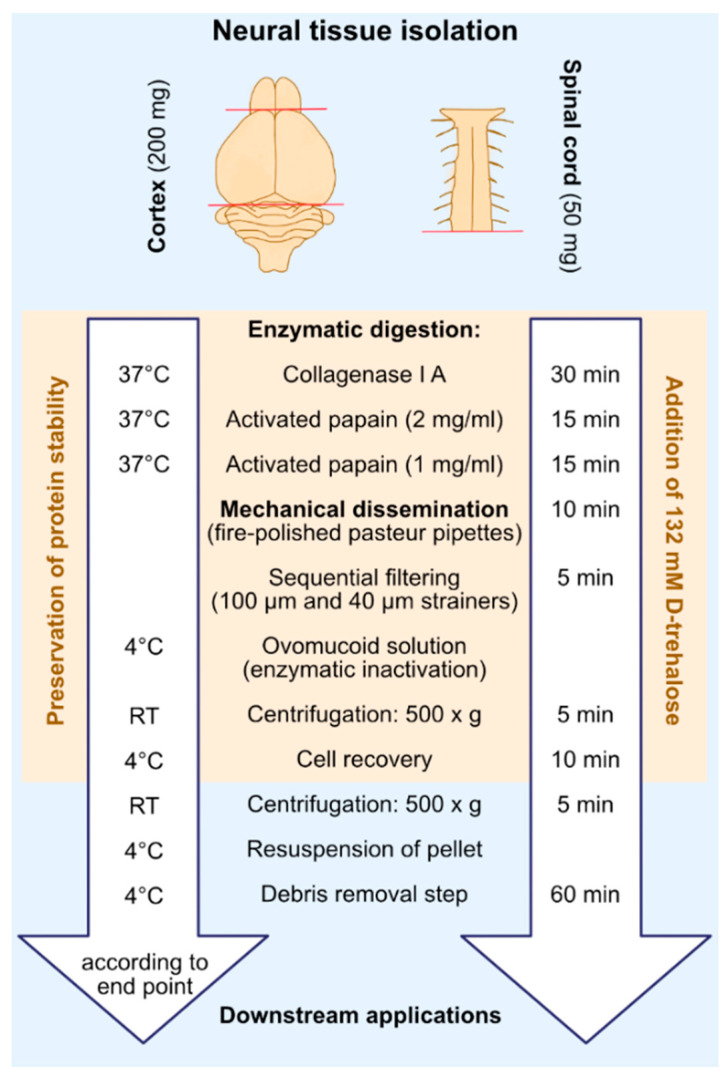
Flowchart delineating key procedural steps of the isolation algorithm. Bilateral murine cortices (~200 mg) or cervical and thoracic spinal cord moieties (~50 mg) are extracted. CNS tissue is digested by sequential treatment with collagenase type IA and activated papain coupled with mechanical trituration. To maintain the integrity of cellular components, D-trehalose is supplemented during enzymatic digestion and the cell recovery step. For downstream flow cytometry-based assays, the extract is cleared from debris. The procedure takes ~2–3 h to attain purified neural cell isolates.

Here, we describe an optimized workflow (key steps shown in Figure 1) for neural single cell isolation, which is innovative in terms of its tailored conceptualization for conditions of CNS aging, senescence, and neurodegeneration, apart from its use in young mature CNS. Second, as the procedure is equally efficient for applications to different anatomic aspects, as demonstrated for brain and myelon, it implies expanded usability by a single procedure. Third, apart from its efficiency regarding cellular yield and viability as discussed in the context of available protocols, it is most comprehensive in the harvest of all the prevailing neural cell compounds inherent to the CNS in their biological proportions [35,36,37], including replicative and post-mitotic moieties. With these achievements, we expect the cellular isolates to be amenable for a variety of scientific downstream purposes, as here exemplified for flow cytometry- and FACS-based assessments of different neural cell types encompassing neurons, microglia, astrocytes, and oligodendrocytes. The implementation of flow cytometry-linked techniques for the analyses and enrichment of peculiar neural cell types remains challenging in comparison, e.g., to blood cells or cells collected from cultures, due to excessive debris released from cellular processes during the cell isolation procedures and the cellular heterogeneity inherent to the brain and CNS.

Moreover, we here approved our protocol for the expanded use in a progeria model and for the detection of senescence-like cellular states in the CNS. Due to the low specificity of SA-β-gal in the CNS context endowed with endogenously high lysosomal activity, we exemplify means for label-free separation of senescent cells based on their dense body load, which represents lipofuscin, in parallel with established morphological criteria [34,38,39]. Such a moiety might be open to be categorized for a specific SASP profile in replicative glia versus post-mitotic neurons, which can be assessed either by secreted factors directly or by the secretome on mRNA levels.

The availability of an optimized neural cell isolation protocol suitable for healthy mature, aging, and neurodegenerative CNS settings complements the current scientific literature, responds to the technical needs in aging and neurodegenerative neurosciences, and thus aims to support our understanding of how the brain ages and how aging, senescence, and age-related pathologies are mutually interconnected. It supplements technical advances dedicated to, e.g., studying CNS morphogenesis and neurodevelopmental disorders [3] and the attempt to realize CNS repair by stem cell-mediated cell replacement [40]. Being appropriate for use in physiologically aged and progeria models, it will provide an integrated cell-based approach to study chronological versus biological aging in parallel with senescence signatures and assist studies addressing age-related phenomapping [19]. Though highlighted here for the brain cortex and spinal myelon, the protocol is expected to comply with other neuroanatomic regions, such as hippocampus, by implementing the appropriate adjustments.

## 2. Results

The bi-hemispheric cortex was harvested from highly aged, asymptomatic C57BL/6 animals serving as a model of physiological, healthy brain aging. Cortices of symptomatic progeroid *Klotho*^−/−^ animals were isolated within the last weeks of their drastically reduced lifespan (see Methods). The cortex, as well as the cervical and thoracic spinal cord segments, were extracted from diseased *hSOD1^G93A^* mutant animals mimicking a phenotype of hereditary ALS. In this model of progressive neurodegeneration, the severity of the motor deficits, elicited primarily by apoptotic death of motor neurons in the anterior horns of the spinal cord, were standardized and assessed according to a clinical deficit score (see Methods, Table 1). Age- and sex-matched mice represented the young control counterparts for the cortex and spinal cord targets of each of the models delineated.

### 2.1. Quality Assessment of Cell Isolates

For quality assessment of the neural cell isolates harvested from an aging and neurodegenerative CNS, tissue preparations were evaluated according to three main parameters: overall cellular yield, vital fraction within the entire isolate, and preserved cell type-specific proportions, with the last two being most efficiently addressed by flow cytometry techniques. As for the progeria model, we focused on the overall cellular output. Apart from an unambiguous flow cytometry-based separation between apoptotic cells, and debris on the one hand and the vital cell population on the other, doublets and other non-singularized cell agglutinations were excluded as a further basic requirement to discriminate target cells at the single cell level. From this starting point of singlets identification (see Methods), which was verified in our hands previously by DNA content analyses with DNA-sensitive dyes, such as DAPI or propidium iodide (PI), in vital and fixed cells, respectively, the target parameters defined above were further confirmed according to the following:

#### 2.1.1. Total Cellular Yield

Cell isolates were based on an innovative and improved strategy optimized by sequential enzymatic tissue digestion and mechanic cell dissociation steps in synergy with the cell and membrane stabilizing properties of the di-saccharide D-trehalose [1,41,42].

**Figure 2:**
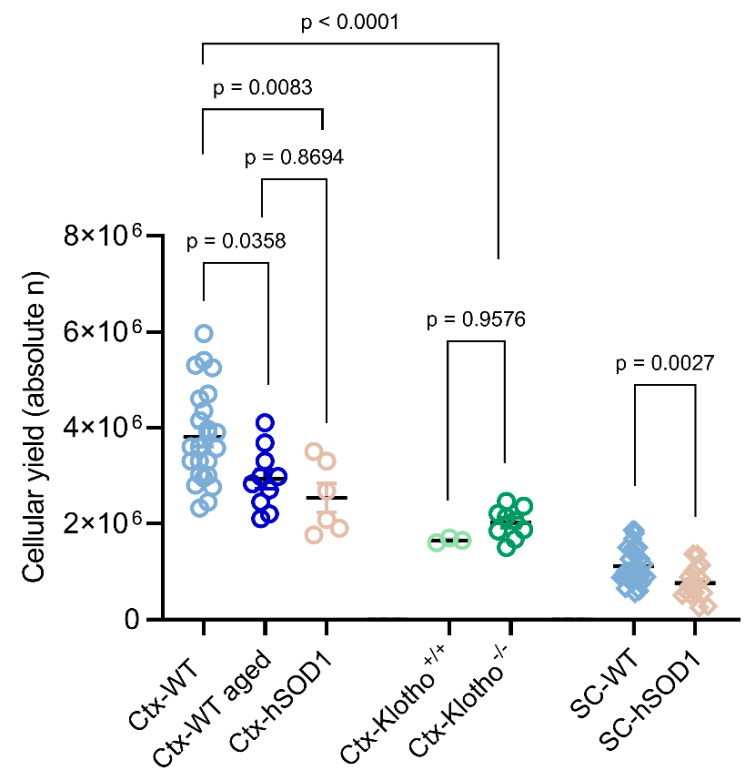
Cellular yield obtained from cortical and spinal cord isolates under healthy mature, chronological aging, progeroid, and ALS-like conditions. Absolute neural cell numbers collected from individual isolates after debris removal step are displayed for young mature (n = 24), wild type aged (n = 10), and *hSOD1^G93A^* mutant (n = 6) cortices, as well as for *Klotho^+/+^* control (n = 3) and progeroid *Klotho*^−/−^ (n = 9) cortices. Cellular yield from spinal cord was assessed from wild type (n = 31) and *hSOD1^G93A^* (n = 17) mutants. For statistical analyses, a one-way ANOVA with post hoc Tukey’s multiple comparison test was performed for cortex and a Student’s t-test was applied for spinal cord. WT, wild type; Ctx, cortex; SC, spinal cord; n, numbers.

As a first quality parameter, the cellular yields were estimated for cortex and spinal cord isolates and compared between young, aged, and neurodegenerative conditions. On average, the debris-purged cellular yield from a bi-hemispheric young mature control cortex comprised ~3.8 × 10^6^ neural cells (Figure 2). Isolates from the young mature wild type spinal cord, comprising the ventral and dorsal portions of the cervical and thoracic segments, provided ~1.1 × 10^6^ neural cells (Figure 2). Physiological aging conditions significantly diminished the overall cellular harvest from cortical tissue by ~23% (Figure 2), equating to an absolute output of ~2.9 × 10^6^ neural cells (*p* = 0.0358; n = 24 for young wild type group; n = 10 for aged wild type group). Likewise, the absolute cellular yield retrieved from the neurodegenerative ALS-mutant cortex was reduced, on average, to ~2.5 × 10^6^ cells (*p* = 0.0083; n = 24 for wild type young cortex; n = 6 for *hSOD1^G93A^* mutant specimens), which equates to an overall relative loss of ~33% (Figure 2). Similar as in cortex, the conditions of ALS-like neurodegeneration led to a compromise of the entire cellular output collected from the spinal cord, resulting in an absolute yield of ~7.6 × 10^5^ neural cells (*p* = 0.0027; n = 31 for wild type young spinal cord; n = 17 for *hSOD1^G93A^* mutant specimens), which accounts for an overall relative reduction in the cellular yield by ~31% in the transgenic as compared to the age-matched control spinal cord (Figure 2).

The total cellular yield of cortical neural isolates obtained from 8–9 weeks old prematurely aged *Klotho*^−/−^ mice was 47% lower than in the 17–21 weeks old wild type C57BL/6 controls (Figure 2; *p* <0.0001; n = 24 for young wild type group; n = 9 for *Klotho*^−/−^ group). Compared to the age-matched 8–9 weeks old controls, the absolute cellular yield achieved from the *Klotho*^−/−^ cortices was slightly, but not significantly, increased by ~22%, attaining an absolute yield of ~2.0 × 10^6^ neural cells (*p* = 0.9576; n = 3 for control group; n = 9 for *Klotho*^−/−^ group; Figure 2). Considering the severe myelination deficits described for *Klotho*^−/−^ mice [43], such a minor increase in the absolute cellular yield might result from a higher efficiency in purging the neurons from myelin components.

In total, the efficiency of the protocol in terms of neural cell retrieval from bi-hemispherical murine cortices (Supplementary Table S1) is comparable to or superior to the previously described protocols established for cell isolations from CNS tissue [1,44]. The application of a debris removal step reduces the collected cell densities by ~50%, however, it was performed in favor of a gain in the purity of intact cells. The purged yield is estimated to be high enough to allow for different downstream applications (graphical abstract), such as FACS, qPCR, and DDR assays, as proven in our own studies [45,46], and might be equally comfortable for other experimental destinations (graphical abstract). The suitability of the cell isolate is, for most of these applications, crucially linked to the proportion of cells preserved in a vital state.

#### 2.1.2. Viability of Isolated Cells

**Figure 3:**
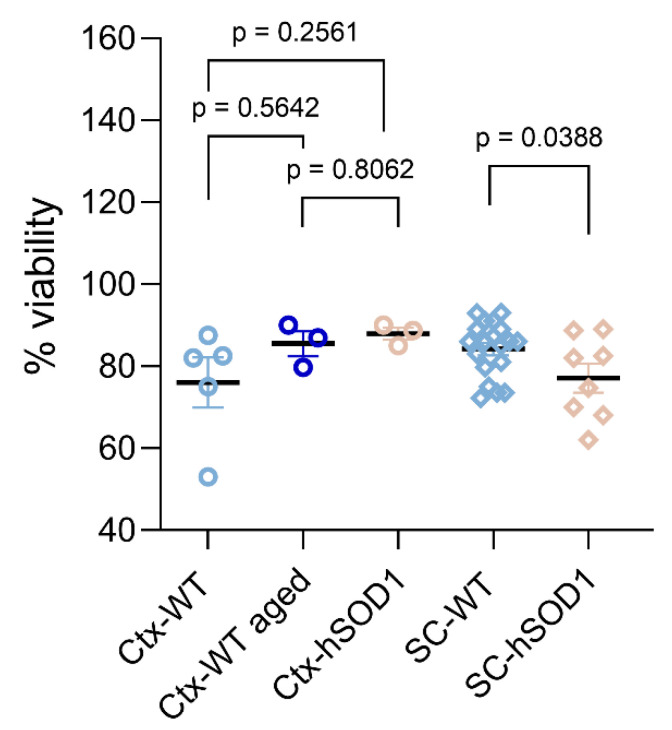
Percent cell viability for cortical and spinal cord isolates underlying wild type, aging, and ALS-like states. Neural cell viability was estimated after exclusion of debris by trypan blue assay and expressed as the percentage of cells surviving the isolation procedure. Cortex: n = 5 for wild type; n = 3 for aging; n = 3 for *hSOD1^G93A^* specimens. Spinal cord: n = 19 for wild type; n = 8 for *hSOD1^G93A^* specimens. For analyses on cortex, a multiple comparison was performed applying one-way ANOVA with post hoc Tukey’s test. For analyses on spinal cord, Student’s *t*-test was applied. WT, wild type; Ctx, cortex; SC, spinal cord.

The viability of isolated cells was determined by standard means by applying the polyanionic azo-dye trypan blue. Bright cells with preserved cellular membrane integrity, as indicated by the absence of a trypan blue cytoplasmic uptake, were considered as vital, intact cells. Vice versa, cells depicted by a trypan blue incorporation due to membrane disintegration, and those appearing with a solid, round morphology, were counted as dead cells. The procedure-related cellular viability estimated for both the cortex and spinal cord was ~80% of the total cell amount harvested after the debris removal step (Figure 3), as demonstrated for the wild type, physiological aging, and *hSOD1^G93A^* transgenic conditions (Figure 3). Thus, it was similarly preserved for the tissue with an increased apoptosis susceptibility (ALS-related mutation; aging) as in healthy circumstances. Though, regarding the spinal cord, the absolute output of vital cells was reduced to a certain degree from ALS mutants as compared to non-transgenic specimens (Figure 3), the consolidated overall viability rate still accounted for approximately 77% (*p* = 0.038; n = 19 for wild type young group; n = 8 for *hSOD1^G93A^* mutant specimens), and thus again provided comparable quality for physiological and degenerative situations. The cellular viability for cortical isolates, independent of the underlying conditions, was >80% and remained without a significant difference between the groups (*p* = 0.5642 for young and aged comparison; *p* = 0.2561 for young and neurodegenerative cortices; n = 5 for wild type young cortex; n = 3 for aging condition; and n = 3 for *hSOD1^G93A^* mutant specimens).

#### 2.1.3. Preservation of Physiological Proportions of Neural Cell Populations

**Figure 4:**
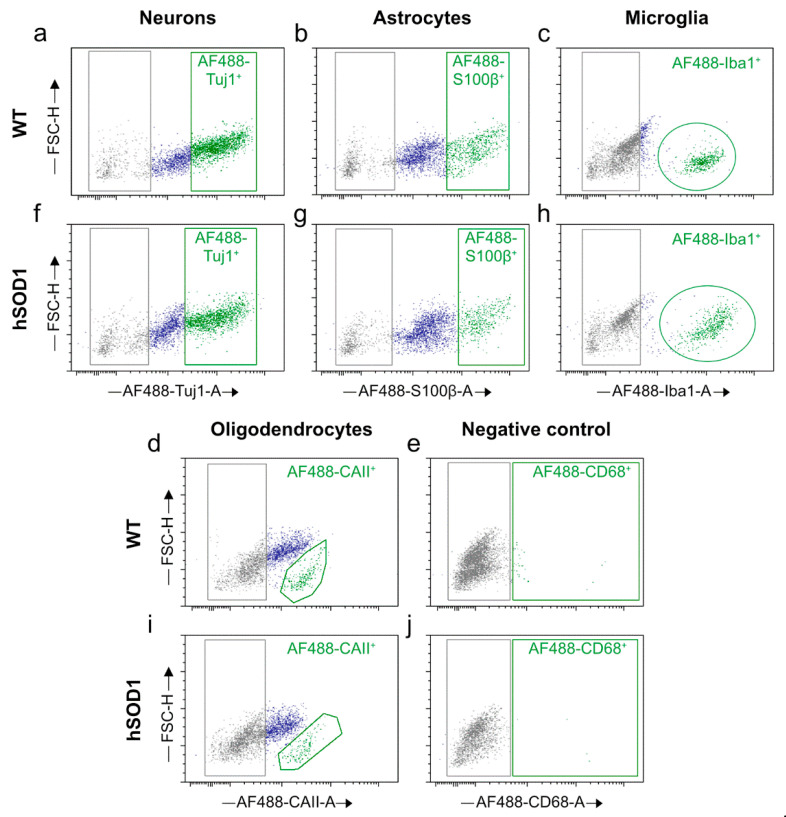
Dot plots representing individual neural cell populations in cervical and thoracic spinal cord isolates. (**a**–**d**) Dot plots for the identification of neural cell entities in wild type animals. (**f**–**i**) Dot plots depicting selection of different neural cell types in *hSOD1^G93A^* mutant animals. (**e**,**j**) Negative control for approval of primary antibody specificity in (**e**) wild type and (**j**) ALS transgenic conditions lacking AF488^+^ events with the CD68 marker, which primarily detects mononuclear cells but spares expression in CNS-autochthonic cell populations. (**a**–**j**) grey colour indicates autofluorescence according to the gating strategy as delineated in the methods section; blue colour depicts singlet cell moieties with intermediate marker affinity; lower signal intensity is considered as background fluorescence. Populations marked in green represent cells with high marker affinity and strong AF488^+^ signal intensity and were assessed as cell type-specific events. FSC-H, forward scatter-height; AF488-A, Alexa Fluor 488-area.

**Figure 5:**
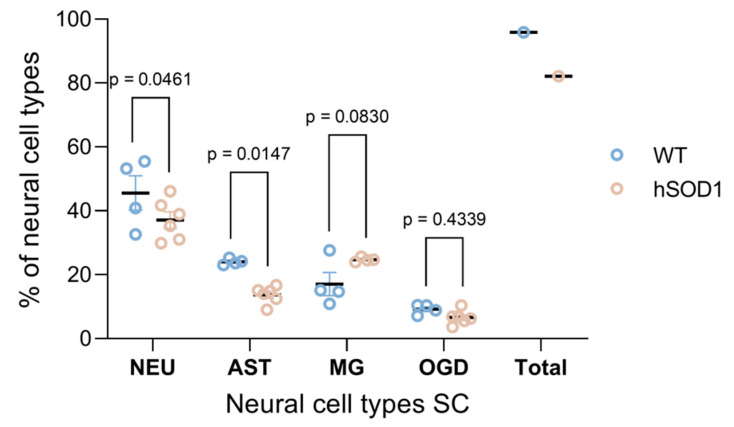
Neural cell proportions in isolates derived from wild type and transgenic spinal cord. Cellular isolates from wild type and transgenic spinal cord were immuno-stained using entity-specific cellular markers. Individual cell types were identified with FACSAria^TM^ Fusion device and assessed via De Novo FCS Express version 5 Plus Flow Cytometry software. For wild type conditions, n = 4. For *hSOD1^G93A^* mutants, n = 4–6. Analyses were performed applying two-way ANOVA with Holm-Šidák’s post hoc test. NEU, neurons; AST, astrocytes; MG, microglia; OGD, oligodendrocytes; WT, wild type; SC, spinal cord.

**Figure 6:**
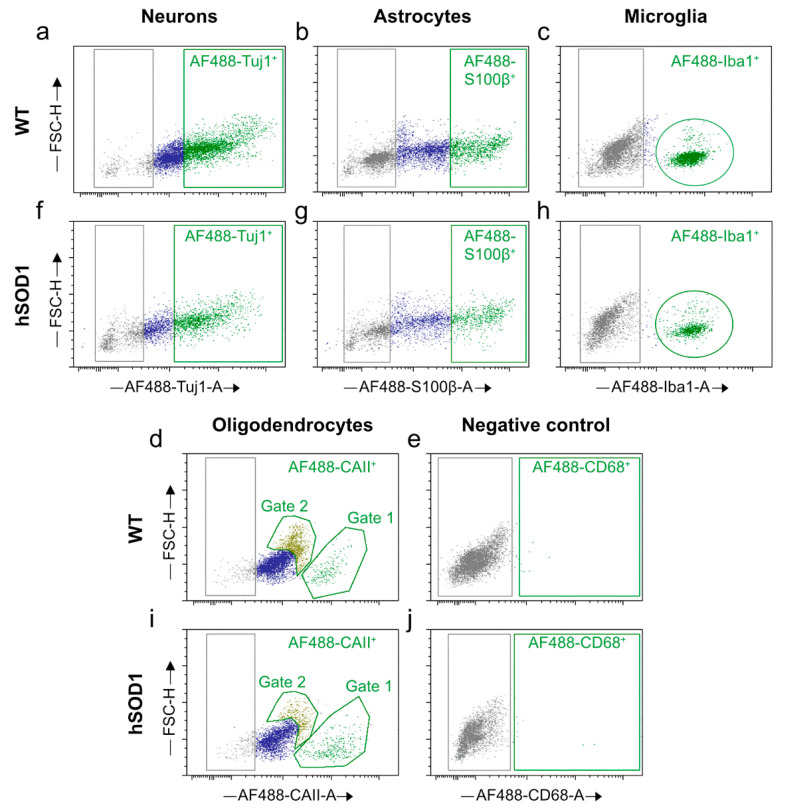
Dot plots representing individual neural cell populations in cortical isolates. (**a**–**d**) Dot plots exemplifying cellular identities in wild type animals. (**f**–**i**) Dot plots illustrating different neural cell types from *hSOD1^G93A^* mutant animals. (**e**,**j**) Negative control for approval of primary antibody specificity (**e**) in wild type and (**j**) ALS transgenic specimens. Note that two oligodendroglial populations are separated both in (**d**) the wild type and (**i**) the transgenic situation. FSC-H, forward scatter-height; AF488-A, Alexa Fluor 488-area.

**Figure 7:**
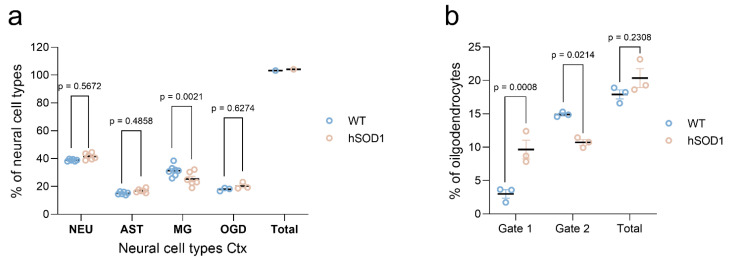
Neural cell proportions in isolates from wild type and transgenic brain cortices. (**a**) Cellular isolates originating from wild type and transgenic cortices were immune-stained using entity-specific cellular markers. Individual cell types were identified with FACSAria^TM^ Fusion device and assessed via De Novo FCS Express version 5 Plus Flow Cytometry software. (**b**) Oligodendrocyte proportions in cortical isolates derived from wild type and *hSOD1^G93A^* mutant animals. Oligodendrocytes clustered in two subpopulations, displaying altered proportions in the diseased state. The fraction in gate 1, characterized by smaller oligodendrocytes with intense CAII signals (Figure 6d,i), gained in numbers, while the moiety in gate 2, featuring lower signal intensities but higher cell size (Figure 6d,i), decreased in quantity as compared to controls. The entire amount remained unchanged in the two groups. For wild type and transgenic *hSOD1^G93A^* mice, n = 3–6 in (**a**) and n = 3 in (**b**). For (**a**,**b**), a multiple comparison was performed by two-way ANOVA with Šidák’s post hoc test. NEU, neurons; AST, astrocytes; MG, microglia; OGD, oligodendrocytes; WT, wild type; Ctx, cortex.

When stratifying the entire cellular yield for the main CNS-autochthonous neural cell entities, i.e., neurons, astrocytes, oligodendrocytes, and microglia, a pattern of well-preserved physiological cell proportions was mirrored in the final isolates either from the spinal cord (Figure 4 and Figure 5) or from the cortex (Figure 6 and Figure 7) both under young mature conditions as well as targeted neurodegeneration. The cellular isolates were stained for different entity-specific markers, such as β-III tubulin for neurons, S100β for astrocytes, Iba-1 for microglia, and CA-II for oligodendrocytes, and the cell moieties positive for the individual cell markers were selected by flow cytometry, followed by the calculation of their proportions for each tissue type and condition. The cell proportions in young versus aged wild type cortices have been recently characterized by means of immunofluorescence using adjusted marker concentrations [45].

##### Cell Proportions for Spinal Cord Isolates

In good accordance, the amounts of neurons harvested from an average absolute yield of ~1.1 × 10^6^ and ~7.5 × 10^5^ cells derived from wild type and *hSOD1^G93A^* mutant spinal cord specimens, respectively, constituted ~45.5% under physiological wild type conditions; however, neurons were reduced to ~37% in severely affected transgenic mice (*p* = 0.0461; n = 4 for wild type young spinal cord; n = 6 for *hSOD1^G93A^* mutant specimens) (Figure 4a,f and Figure 5). Correspondingly, astrocyte composition diminished from ~24% in healthy animals to ~13.7% in animals with underlying neurodegeneration (*p* = 0.0147; n = 4 for wild type young spinal cord; n = 6 for transgenic specimens) (Figure 4b,g and Figure 5). In contrast, microglial populations underwent an expected incremental increase from ~17.0% under naïve conditions to ~24.7% under transgenic conditions, though this increment did not reach significance (*p* = 0.0830; n = 4 for both wild type young and *hSOD1^G93A^* mutant specimens) (Figure 4c,h and Figure 5). Though out of the scope of this protocol, a further differentiation addressing the abundances and the transformation of M1 into M2 phenotypes might be beneficial, in case pro-inflammatory versus repair effects are to be characterized. Oligodendroglia moieties remained unchanged, providing values of ~9.20% and ~6.59% for wild type and transgenic mice (*p* = 0.4339; n = 4 for wild type young spinal cord; n = 6 for *hSOD1^G93A^* mutant spinal cord), respectively (Figure 4d,i and Figure 5). These numbers account for a vital moiety purified from cellular and nuclear debris and apoptotic cells. For the neurodegenerative spinal cord, a considerable loss of neurons with expected affection of motor neurons, the most vulnerable population in the ALS pathology, was evident along with this process of cell damage and loss; the tendential increase in immune response-mediating and phagocytically active microglial populations might reflect the pro-inflammatory status implicated in the ALS pathophysiology. The specificity of the marker-related sorting events was verified by the CD68 epitope, which lacks expression in CNS autochthonous cell populations and thus served as a negative control (Figure 4e,j).

These findings underline the sensitivity of our cell isolation protocol to reveal dynamics in the relations of cellular densities that often parallel functional deterioration and disease progression.

##### Cell Proportions for Cortical Isolates

According to the predominantly spinal rather than cortical motor neuron pathology characteristic of our ALS model, neural cell isolates from the cortex manifested a comparable neuronal yield of ~38.9% from the wild type and of ~41.5% from the mutant animals, respectively (*p* = 0.5672; n = 6 for both wild type young and *hSOD1^G93A^* mutant specimens) (Figure 6a,f and Figure 7a). In a similar manner, ~15.1% and ~16.9% of vital cells exhibited astrocytic identity in the wild type and transgenic group, respectively (*p* = 0.4858; n = 6 for wild type and mutant cortices) (Figure 6b,g and Figure 7a). Unexpectedly, with ~25% of the neural cells identified to be microglia, the transgenic cortices showed a lesser amount of microglia as compared to the ~31% detected under naïve wild type conditions (*p* = 0.0021; n = 7 for wild type young cortex; n = 6 for *hSOD1^G93A^* mutant specimens) (Figure 6c,h and Figure 7a). Previously, the neural cortical isolates from the young and aged wild type animals exhibited an average microglia percentage of ~14.0% [45]. The current robust level of microglial entities in the naïve wild type cortex appears well-explained by the optimized procedural purification of the isolate and also accounts for the high sensitivity of flow cytometry-based cell identifications, which are often superior to immunofluorescence microscopy and thus might have detected dynamic M1 and M2 phenotypes with improved thresholds. In how far a dampening of M2 moieties might explain the reduction of the total microglia population isolated under ALS-like neurodegeneration might be further addressed by polarization framework analyses.

The total amount of oligodendrocytes under both conditions remained unchanged, as depicted by the percentages of ~17.90% and ~20.36% for the wild type and mutant *hSOD1^G93A^* animals, respectively (*p* = 0.6274; n = 3 for both wild type young and *hSOD1^G93A^* mutant specimens) (Figure 6d,i and Figure 7a). Following spinal cord analyses, the CD68 marker served as a negative control in the cortex (Figure 6e,j).

When selecting for oligodendrocytes in cortical isolates, a partitioned appearance in the dot plots was observed, as indicated by gate 1 and gate 2 in Figure 6d,i. While the moiety in gate 1 represents oligodendrocytes of higher signal intensity for the CAII marker (AF488 CAII-A), those clustered in gate 2 are increased in size as indicated by the high FSC-H index (Figure 6d,i and Figure 7b). When comparing these populations under wild type and neurodegenerative conditions, an increment of the moiety in gate 1 at the expense of the gate 2 population was evident, while the entire amount remained without significant differences (Figure 7b).

Thus, the neural cell isolation protocol introduced does not only stratify for individual neural cell entities but is even sensitive to select for sub-entities within a defined cellular population. The interesting issue as to whether these changes in oligodendrocytic features detected by flow cytometry are related to a process of oligodendroglial neogenesis and the appearance of precursor states, or other morphological changes related to the ALS-based degeneration process, still has to be elucidated. Likewise, the mature CNS harbors morphologically and functionally heterogeneous oligodendrocyte populations, which segregate with spatial preferences, distinguish in their natural survival times, and respond with different vulnerability to traumatic stressors [47,48]. Moreover, adaptive myelination found during the process of fibers tract maturation is indicated to continue into adulthood and to allow for plasticity in response to injury [48]. Thereby, some oligodendrocyte subtypes might be particularly prone to drive remyelination, repair, and adaptive plasticity, though their spatial prevalence might be preserved even in the chronic phase of Wallerian degeneration [47,48].

In a very recent protocol designed for -omics studies on neural nuclei isolated from human and murine CNS, a total of ~3 × 10^6^ neuronal cells, ~1.8 × 10^6^ oligodendrocytic nuclei, and ~3–4 × 10^5^ myeloid/microglial cells were harvested from the whole brain of 8–10-week-old C57BL/6 animals [49]. Though roughly doubled in absolute quantities as compared to our cortical output accounting for ~1.6 × 10^6^ neurons, ~1.3 × 10^6^ microglia, and ~7.2 × 10^5^ oligodendrocytes (and ~6.3 × 10^5^ astrocytes; for details regarding absolute cellular yields, see Supplementary Tables S2 and S3), the proportions of the cell fractions purified are highly comparable and correspond to the moieties achieved with our protocol. The ~50% reduction in the absolute yield of the neuronal and oligodendrocyte fractions to be compared is most likely due to the difference in the anatomic target areas as the amounts achieved by Nott and colleagues [49] are based on an entire frozen murine brain, whereas the numbers shown here are restricted to freshly isolated murine bi-hemispherical cortices. Similarly, our data well correspond to the isolation of, e.g., ~2.5 × 10^5^ vital microglia from the whole brain as reported for 8–9 weeks old C57BL/6 mice, with a mechanical dissociation technique assisted by a Percoll gradient [50]. In line with such data, an own previous precursor variant of our herewith introduced protocol coupled to a Percoll gradient and density centrifugation step gathered ~5.0 × 10^5^ and up to ~1.0 × 10^6^ microglia from murine cortices. Moreover, while our protocol is suspicious to underrepresent the oligodendroglial moiety in favor of better purification from debris, including myelin-derived cell and membrane particles, the data from Nott and colleagues may putatively underestimate the fraction of microglia within the myeloid cells in their isolate [49].

### 2.2. Autofluorescence Pattern in Young, Aged, Progeroid, and Neurodegenerative Specimens

Endogenous, tag-free autofluorescence has been used as a real-time measure to assess the senescent state of cultured cells and correlate them with the aging state of tissues, which owes to their high lysosomal content and the increase in the intra-lysosomal load of indigestible lipofuscin deposits [39,51]. Thus, being considered as signatures of cellular senescence, autofluorescence (AF) in cortical and spinal cord samples from aged, progeroid, and *hSOD1^G93A^* mutant animals and appropriate young controls was assessed by flow cytometry under simultaneous application of a green B530 and yellow/green YG582 laser. As apoptotic cells are reported to be reduced in autofluorescence [51], only the vital singlets were assessed.

**Figure 8:**
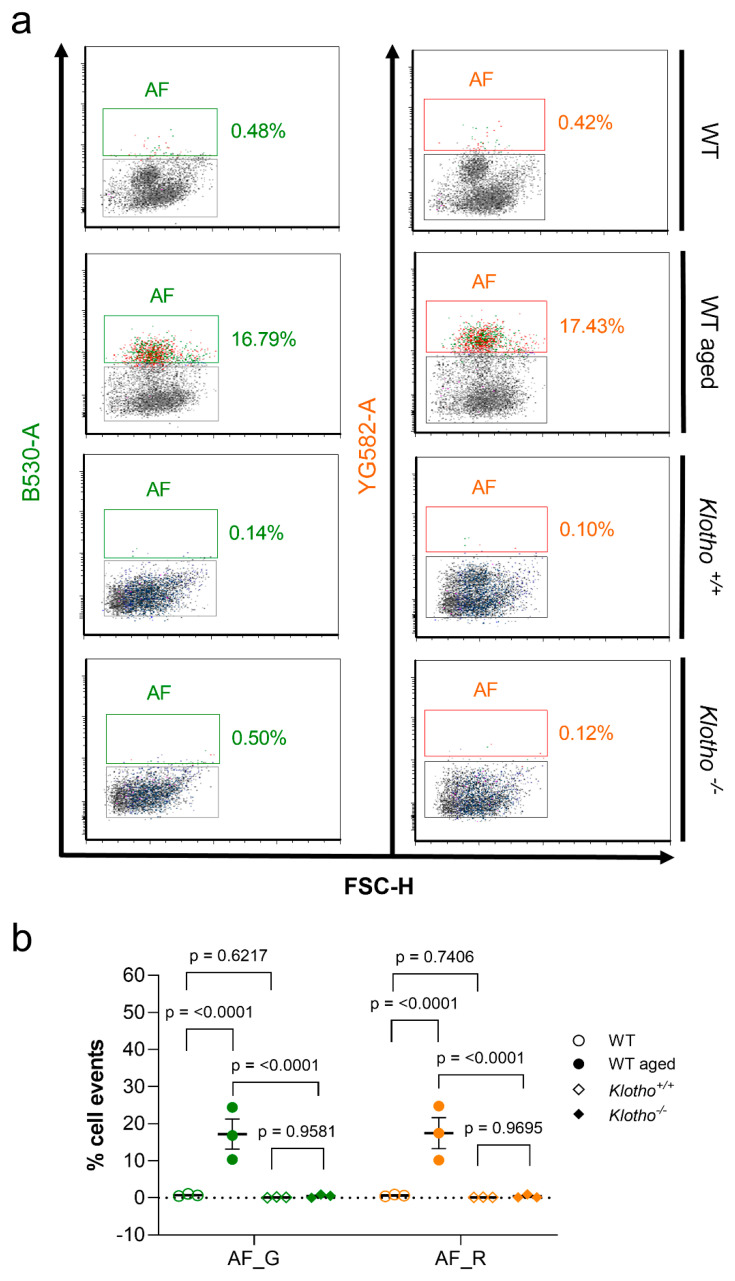
Cellular autofluorescence exemplified for cortex under wild type aged and progeroid conditions in comparison to respective controls. (**a**) Autofluorescence (AF), indicated by broad signal emissions both from the green (B530) and yellow/green (YG582) channels, was low in young wild type control specimens (**a**, upper row) but drastically increased in cellular isolates from aged counterparts (**a**, second row). By contrast, specimens from *Klotho*^−/−^ animals at the stage of severe symptoms remained without an accumulation of AF cells (**a**, bottom row), which showed a comparable frequency in *Klotho^+/+^* controls (**a**, third row). (**b**) Quantitative analyses of cells emitting AF, separated for B530 and YG582 channels. n = 3 per group and parameter. For b, a multiple comparison was performed by two-way ANOVA with Tukey’s post hoc test. B530-A, B530-area; YG582-A, YG582-area; AF_G, autofluorescence in green B530 channel; AF_R, autofluorescence in yellow/green YG582 channel; FSC-H, forward scatter-height; WT, wild type.

**Figure 9:**
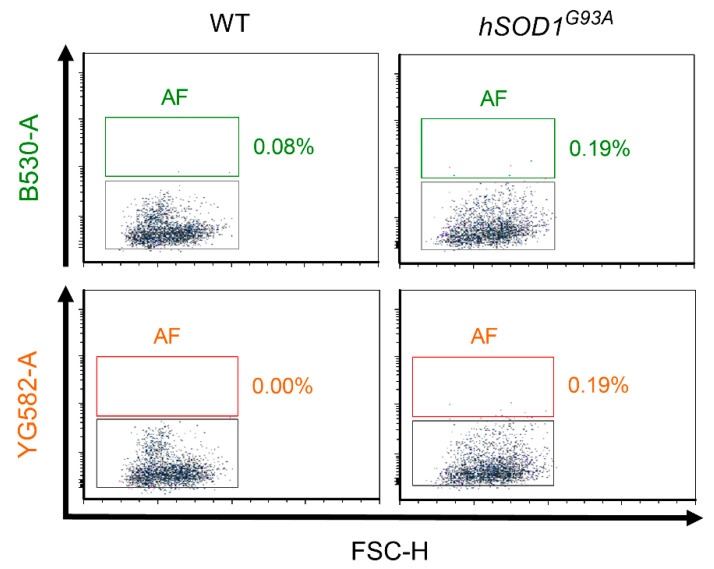
Cellular autofluoescence exemplified for spinal cord under neurodegenerative *hSOD1^G93A^* and young wild type control conditions. Autofluorescence (AF), indicated by simultaneous, broad signal emissions from the green (B530) and yellow/green (YG582) channel, in specimens derived from symptomatic *hSOD1^G93A^* mutant animals was indistinguishable from control isolates in both channels. AF, autofluorescence; FSC-H, forward scatter-height; WT, wild type, B530-A, B530-area; YG582-A, YG582-area. n = 3 per group and parameter.

We observed a remarkable increase in AF, indicated by a broad, simultaneous signal emission from the B530 and YG582 channels, in highly aged cortical specimens. Under aging conditions, 17.16 ± 4.05% and 17.43 ± 4.21% of the singlet cells were found as a source of AF in the B530 and YG582 channel, respectively, accounting for a 23.8-fold and 27.6-fold increase over the average of 0.72 ± 0.17% and 0.63 ± 0.13% AF emitted from the B530 and YG582 channel in young cellular states (*p* = 0.0154 for the B530 channel; *p* = 0.0162 for the YG582 channel; n = 3 for each condition; Figure 8a,b). By contrast, AF was negligible in the progeroid *Klotho*^−/−^ model, accounting for 0.45 ± 0.25% and 0.38 ± 0.26% of the singlets in the B530 and YG582 channel at the stage of severe precocious aging symptoms and was not increased over control levels (0.14 ± 0.07% AF in the B530 channel, *p* = 0.2926; 0.14 ± 0.02% AF in the YG582 channel, *p* = 0.400; n = 3 both for *Klotho*^−/−^ and aged-matched controls; Figure 8a,b). Notably, autofluorescence in *hSOD1^G93A^* mutant neurodegenerative spinal cord samples was also not elevated, but remained comparable to that emanating from young controls (0.05 ± 0.03% and 0.07 ± 0.07% versus 0.14 ± 0.04% and 0.08 ± 0.05% in the B530 and YG582 channels of young and *hSOD1^G93A^* mutant specimens, respectively; for the B530 channel, *p* = 0.128; for the YG582 channel, *p* = 0.889; n = 3 for both young control and *hSOD1^G93A^* mutant specimens; Figure 9).

### 2.3. Stratification of Cells Responsible for Enhanced Autofluorescence in Highly Aged Specimens

**Figure 10:**
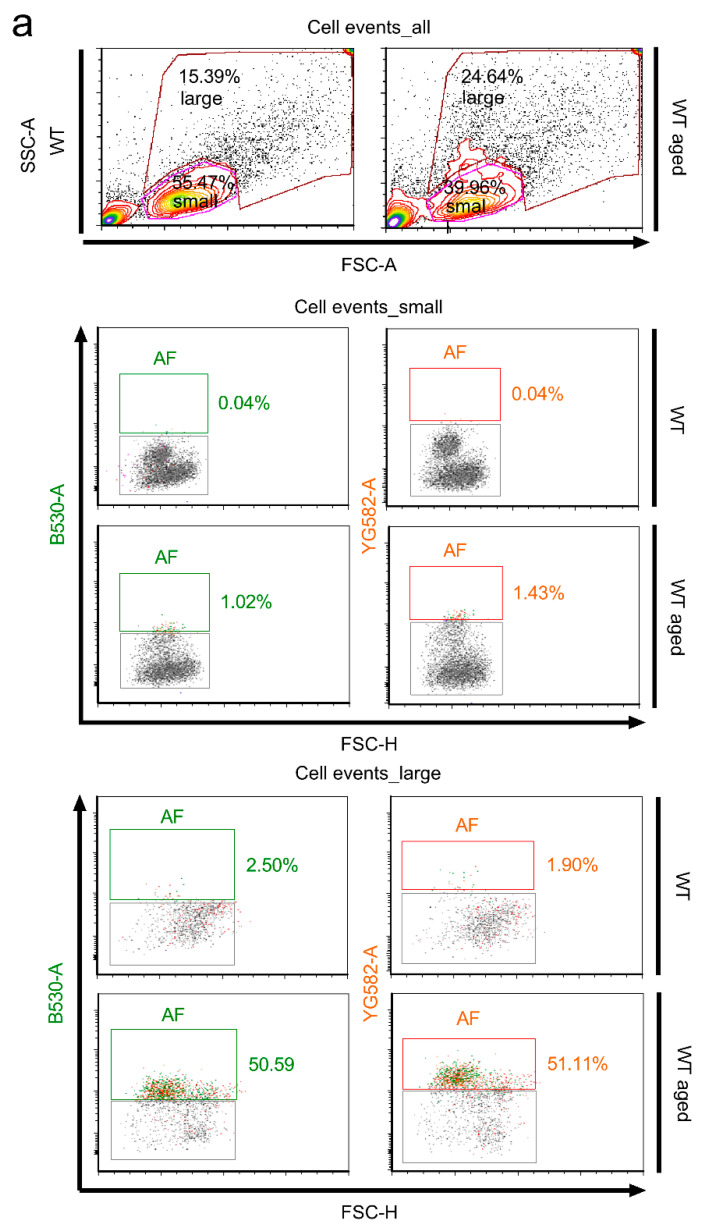

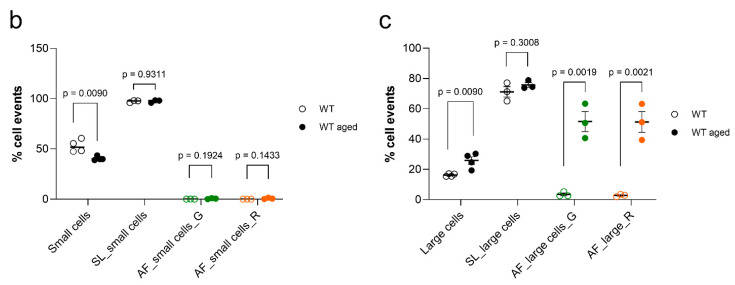
Cellular autofluorescence exemplified for young versus aged wild type cortical specimens. (**a**) The total amount of vital cellular events harvested by flow cytometry, excluding apoptotic moieties, was size-separated into a condensed cluster of small cells and a scattered population of large-size cells (top row in **a**). Gating against autofluorescence (AF), i.e., displaying a typical ubiquitous emission from both the green (B530) and yellow/green (YG582) channels, indicated a drastic age-dependent rise in AF signals (**a**, second row in last block) as compared to young specimens (**a**, first row in last block). The data illustrate that the vast majority of AF cells originate from large-size cells (last block) with increased granularity, rather than from small-size cells (middle block) as assessed by FSC-A and SSC-A parameters, irrespective of age. (**b**,**c**) Quantitative discrimination of the total amount of sorted cellular events, according to the following parameters: total of small (**b**) and large cells (**c**); singlets (SL) of small (**b**) and large (**c**) moieties; singlets displaying AF in green B530 (SL_G) or yellow/green YG582 (SL_R) channel in either the small-size (**b**) or large-size (**c**) population. AF, autofluorescence; SL, singlets; WT, wild type; FSC-A, forward scatter-area; FSC-H, forward scatter-height; SSC-A, side scatter-area, B530-A, B530-area; YG582-A, YG582-area. n = 3–4 per group and parameter. For (**b**), a multiple comparison was performed by two-way ANOVA with Tukey’s post hoc test.

Apart from autofluorescence, senescence is characterized by morphological changes such as flattened and increased soma size and higher cellular granularity [38,39,51]. Therefore, autofluorescence was co-assessed for these parameters by flow cytometry, engaging the forward scatter area (FSC-A) and side scatter area (SSC-A) as measures of size and granularity, respectively. Based on the dot plot characteristics illustrated in Figure 10, the overall quantity of vital singlets sorted from the entire population of cortical cell events was gated into two moieties, providing a dense cluster of ‘small cells’ of lower granularity and a scattered cloud of ‘large cells’, displaying an enlarged soma size and augmented granularity indicative of a senescent cell transformation (Figure 10a). The cell portion exhibiting this senescence-associated phenotype increased in highly aged specimens as compared to young wild type specimens, as exemplified in Figure 10a (25.75 ± 2.5% in aged versus 16.21 ± 0.45% in young samples, *p* = 0.0090, n = 4 for each group), while the portion of ‘small cells’ was reduced from 52.96 ± 3.11% in young specimens to 40.55 ± 1.02% in aged specimens (*p* = 0.0090, n = 4 for each group, Figure 10a,b). These results reflect the phenotypic alterations entailing the aging process. Notably, the small-sized cells devoid of phenotypical characteristics of senescence lacked substantial AF emission, irrespective of whether they were assessed under young (0.07 ± 0.03% and 0.06 ± 0.01% AF singlets in the B530 and YG582 channel, respectively) or aged conditions (0.52 ± 0.29% and 0.73 ± 0.37% AF singlets in the B530 and YG582 channels, respectively) (Figure 10a; *p* = 0.1924 for the B530 channel; *p* = 0.1433 for the YG582 channel; n = 3 for both groups). By contrast, the cellular moiety gated into the category ‘large cells’, which displays increased granularity, overlapped with 51.53 ± 6.57% and 51.23 ± 6.87% of the events classified as AF emission from the B530 and YG582 channel, respectively, thereby indicating a 15.2-fold and 18.76-fold increase over the 3.39 ± 0.89% and 2.73 ± 0.45% for the control baseline AF (*p* = 0.0019 for the B530 channel; *p* = 0.0021 for the YG582 channel; n = 3 for both groups; Figure 10a,b).

### 2.4. Quenching of Autofluorescence

**Figure 11:**
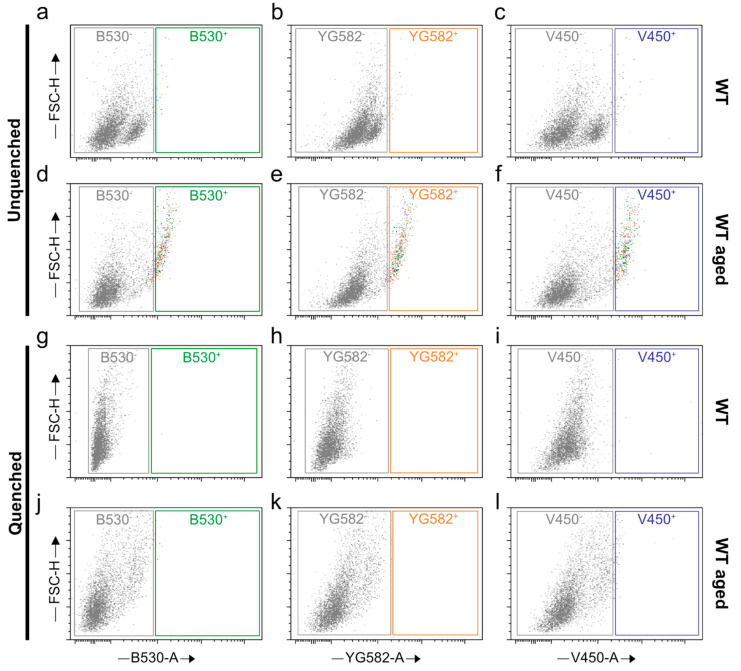
Autofluorescence quenching in young and aged cortical specimens. (**a**–**f**) Dot plots representative of unquenched (**a**–**c**) wild type young and (**d**–**f**) aged specimens. (**g**–**l**) Dot plots for (**g**–**i**) wild type young and (**j**–**l**) aged samples displaying the effect of autofluorescence quenching in B530, YG582, and V450 channels that detect green, red, and violet fluorescence signals, respectively. FSC-H, forward scatter-height; B530-A, B530-area, YG582-A, YG582 area, V450-A, V450-area.

Higher levels of lipofuscin-related autofluorescence in aged specimens can become highly critical with regard to further downstream studies, e.g., if fluorescent measures for specific marker expression are involved. Therefore, we introduce here a quenching strategy that has the capability to specifically suppress lipofuscin-related AF signals. The efficiency of this step is high, as depicted in Figure 11, according to the successful elimination of AF emission from all, i.e., red, green, and violet, channels (Figure 11j–l).

## 3. Discussion

Research on molecular and cellular mechanisms in aging, senescence, and age-associated neurodegenerative diseases requires the use of reproducible and suitable cell isolation protocols [11]. The possibility of extracting all the main CNS-resident cell types from one single tissue allows for specific multi-omic analyses of complex cellular interactions ex vivo and the phenotyping of cell cycle-dependent classical and atypical senescence. Currently available isolation protocols combine multi-dimensional approaches, including RNA sequencing analyses and mass spectrometry, to define cellular profiles [52]. However, the application of such technical advances is often cost-intensive, time-consuming, and dependent on special technical skills [11]. An additional hurdle for efficient neural cell isolation is represented by profound aging-related alterations in the structural and molecular properties of different cellular components, including their membranes [20]. These changes can influence the biophysical and biochemical properties of cells and tissues [20,21,22,23] and interfere with the established stages in the isolation algorithms. Furthermore, isolation of aged CNS neurons is complicated by their intrinsic sensitivity to mechanical stress or chemical exposure, steps that are unavoidable for disintegration of cellular connections and single cell extractions.

The working procedure described here allows for the recovery of viable neural cells with high yield from both adult and aged CNS under physiological and pathophysiological conditions. Recent cytological analyses further confirmed structural integrity at the single cell level [45]. Herewith, it responds to and complies with the peculiar cellular conditions encountered in aged, progeroid, and neurodegenerative tissues. Methodically, it is based on the use of precisely performed stepwise temperature regulated multi-enzymatic digestion of the extracellular collagen matrix. These steps are complemented by means of moderate mechanical trituration and essentially performed in the presence of an osmolytic cell and conformational protein stabilizer. Such a line of action synergizes a series of favorable factors that have been crystallized in bits and pieces in recent publications [1,41,42], thereby aiming for their useful multiplexing in a single, quick and easy-to-perform protocol and the targeted expansion of its application to the peculiar conditions of CNS aging, progeria, and neurodegeneration.

One critical aspect in the achievement of this targeted expansion to conditions of aging and neurodegeneration relies on the application of the homo-disaccharide D-trehalose, a direct cellular membrane and protein stabilizer. Physiologically, in unicellular organisms, trehalose is secreted as part of a stress response elicited by environmental discomfort, e.g., through exposure to thermal stress or low humidity, in order to preserve cellular integrity [53]. Moreover, it has achieved broad applications due to its favorable properties regarding osmoregulation, cryoprotection, and desiccation tolerance [54,55]. According to current biochemical models, trehalose is assumed to either build a protective, cocoon-like matrix around nearby protein structures, to entrap water molecules that are remote from protein structures and reduce protein–water interferences, or stabilize them by the establishment of hydrogen-bonds substituted from water molecules [56]. A similar effect on the replacement of hydrogen bonds from water molecules is indicated for its protective function on membrane phospholipids [53]. The biological importance of trehalose for the structural and functional preservation of protein and lipid structures is also reflected in the context of neurodegenerative conditions. Likewise, trehalose treatment has been evidenced to stabilize aggregate-prone polyglutamine tracts, as exemplified by an engineered, repeat-containing myoglobin protein in vitro, and to reduce protein aggregates by oral treatment in vivo, thereby alleviating motor deficits and improving survival in a mouse model of Huntington’s disease [57]. Moreover, trehalose operates as a DNA protectant of freeze-dried (lyophilized) somatic cells [58] and reduces irradiation-induced single strand breaks in a dose-dependent manner, putatively through free radical scavenging and anti-oxidative properties on chromatin [58,59].

Regarding senescence studies, the fingerprints of a senescence-like state in post-mitotic cells including neurons [28,60] are still not confirmed, but might resemble the cellular and molecular features inherent to the process of replicative senescence [29,61]. Likewise, neurons are consistently exposed to elevated levels of oxidative stress due to their high metabolic activity, while restricted in DNA repair mechanisms. Such genomic stress, which can elicit a persistent DDR that is also in protected telomere regions, propagates senescence [28,62] and is understood as a driving mechanism of aging [63]. In support, we have recently also shown that post-replicative CNS neurons are subjected to age-related telomere length alterations, a key process in replicative senescence [45]. Moreover, post-mitotic CNS neurons can quit their G_0_-arrest and re-enter G_1_/S/G_2_ phases without completing cytokinesis [64,65] and thus adopt DNA content alterations that activate the DDR and culminate in dysfunction, senescence, or apoptosis [61]. Therefore, while the concept of classical senescence implicates an irreversible cell cycle arrest, PoMiCS in neurons are theorized to engage the opposite, i.e., a kind of replicative reprogramming and cell cycle re-uptake [32,61]. Such interference renders the identification of senescence-like processes even more complex, as it excludes the suitability of cell cycle- and DDR-sensitive p21^CIP1/WAF1^ and p16^INK4A^ kinases as early and late senescence biomarkers, respectively. Moreover, the suitability of the extensively applied biomarker SA-β-gal is also restricted due to its low specificity in an environment with intrinsically high lysosomal content, as it serves as a surrogate marker for lysosomal activity [39,66,67]. Moreover, the catalogue of PoMiCS markers might overlap with aging markers. Recently, Piechota et al. suggested the REST protein, or repressor element-1 silencing transcription factor, as a new option for the detection of neuronal senescence [67]. In the context of this protocol, we used endogenous autofluorescence, mainly generated by intracellular lipofuscin deposition, as a selection criterion and showed that autofluorescent cells share morphological features characteristic of senescent cells [39,68]. These signatures include a larger, hypertrophic soma and increased cellular granularity, e.g., due to lipofuscin dense bodies, glycogen granules, or subcellular organelles, and are assessable by sorting analyses [38,51]. While they were found to increase under chronological aging, but not in progeria and *hSOD1^G93A^* mutants, we further illustrate the capacity of this protocol to distinguish between the processes of biological aging, neurodegeneration, and senescence.

Therefore, the described protocol might be profitable in the identification of senescent cells in the CNS and, by further specifications based on targeted cellular and molecular markers, help dissect neuronal PoMiCS from classical senescence. With broader utility, it may also provide a platform for the evaluation of key pathophysiological domains relevant in the process of senescence, thereby encompassing cell cycle status, DNA integrity, and downstream SASP secretory or transcriptomic profiles, and might also allow for assessments on epigenetics.

In terms of output parameters, we achieved, in comparison to our own experiences with previous protocols [1,41], beneficial effects as for the following criteria: (1) enhanced elimination of tissue clumps and improved cell dissemination into singlets at optimized cell viability rates; (2) improved preservation of nuclear integrity; and (3) reduced amounts of fragmented nuclei consecutive to mechanical tissue dissociation [45]. Likewise, Eide and McMurray described a yield of ~7.4 × 10^5^ neural cells underlying a papain-mediated dissociation of striatum or cortical tissue of 1–1.5-year-old mice, sparing any gradient purification step [44]. In comparison, from bi-hemispherical cortices, our protocol results in recovery yields of ~7.2 × 10^6^ and ~6.2 × 10^6^ neural cells obtained from 20–22 and 94–97 weeks old C57BL/6 mice, respectively, with counts being collected in the absence of a debris removal step (Supplementary Table S1). The efficiency attained is comparable to the yield of 6.9 × 10^6^ cells previously harvested from the cortex of 26-week-old mice underlying papain digestion and OptiPrep-gradient centrifugation steps, which help to exclude crude debris particles, as reported by Brewer and Torricelli [1]. Considering such procedures, and additional purging from debris-forming cell fragments and axonal projections, our protocol resulted in the isolation of up to ~1.1 and ~3.8 × 10^6^ neural cells from wild type conditions, given that one spinal cord, comprising of cervical and thoracic segments, or a bi-hemispheric murine cortex was used, respectively. With D-trehalose supplementation, Saxena and colleagues improved the viability of FACS-purified neurons from 58.1% to 81.0%, which is comparable to our herein described achievements [42]. Conditions of aging and ALS-mimicking neurodegeneration were accompanied by an expected decline in the absolute cellular yield. However, the procedure-associated cellular viability estimated for both the cortex and spinal cord was ~80% of the total, debris-free cellular harvest, irrespective of the underlying wild type, aging, or transgenic condition, and thus was similarly preserved for tissues with increased apoptosis susceptibility as it would be for healthy CNS tissue. The cellular output parameters were also specified for the *Klotho*^−/−^ progeria model and compared to standard young C57BL/6 and age-matched *Klotho^+/+^* genotypes. Additionally, the relative proportions of the different neural cell types isolated were preserved under neurodegenerative conditions and thus equalized to a physiological cell composition established for young and aged tissue specimens [35,36,37,45].

In a very recent study, Schroeter and colleagues described a protocol for the isolation of a complete profile of CNS resident cell types in mice, applied either on healthy young CNS or a model of experimental autoimmune encephalomyelitis (EAE) resembling the pathophysiology of multiple sclerosis [11]. Using magnetic activated cell sorting in the frame of a commercial kit approach, the authors obtained a yield of ~5.4 × 10^6^ and 4.0 × 10^6^ cells from the entire brain of normal and EAE-diseased animals, comprising an absolute neuronal yield of 4.5 × 10^5^ and 4.4 × 10^5^ cells, respectively [11]. The oligodendroglial yield accounted for 3.2 × 10^6^ and 2.9 × 10^6^ cells in healthy and EAE-elicited animals, respectively. The enrichment of cellular subtypes, suggested to arise as result of magnetic separation, might indeed be intentionally beneficial for certain target analyses. With our protocol, we collected numbers of neural cells that reflect the proportions described for the cortex and spinal cord under physiological conditions and for neurodegenerative mouse models (Supplementary Tables S2 and S3) [35,36,37].

Thus, this protocol is optimized and tailored for use on healthy and mature physiologically and precociously aged tissues, as well as degeneration-prone murine brain and spinal cord tissues. Accordingly, we see usefulness for a broad repertoire of genetically engineered mice carrying disabling mutations such as in the *Klotho* locus [69,70] and *XPF* and *ERCC1* deficiencies [71], or those developing an ALS-like phenotype due to loss-of-function mutations corresponding to *SOD1, TDP-43,* and *FUS* mutations or *C9ORF72* repeat expansions in humans [72,73,74,75]. Moreover, it is assumed to be useful for the characterization of senescence processes in the CNS. Methodically, apart from our own previous applications in the context of Flow-FISH- or qPCR-based telomere length measurements in the young versus aged CNS [45], the isolation and culturing of physiologically aged microglia [76], and for molecular studies on DNA strand break detection under ALS-like neurodegeneration [46], we expect this protocol to be profitable for further scientific destinations (graphical abstract). These destinations might include cell type-specific culture systems, underlying, e.g., the use of entity-specific vital dyes in combination with FACS strategies such as Neurofluor™ NeuO-mediated neuron selection [45], or the implementation of bead-supported or gradient-based cell separation strategies. Such expansion will allow for the omission of a cellular fixation step and thus render the protocol amenable for RNA profiling and sequencing at an -omic scale. Moreover, by providing a platform for aging studies, it offers single cell access to all cellular identities prevailing in the CNS, thereby approximating physiological cell proportions [35,36,37]. In conclusion, this protocol responds to the growing scientific need in neurosciences to combine ex vivo platforms with the capacities of genetic engineering realized in genetically tailored animals and thus allow for bidirectional study designs that approach from organism-to-cell and are back-verified from cell-to-organism using identical genetic conditions. Such approaches are promising to support a better understanding of age-related neuropathologies and senescence and open up novel targets for therapeutic interventions.

## 4. Materials and Methods

### 4.1. Acquisition of Biological Material

Conditions of neurodegeneration were recapitulated in a well-established transgenic mouse model of ALS, based on the overexpression of a mutant human superoxide dismutase (*hSOD1^G93A^*) gene [77]. The ALS-associated clinical phenotype was monitored in a standardized fashion by applying an investigator-neutral clinical score (Table 1), complemented by preceding and paralleling weight as well as overall health state assessments. Mutant male animals were acquired at an age of 17–21 weeks when they were revealing the onset of motor impairments and the manifestation of a severity level corresponding to a clinical score of at least ‘1′ (Table 1). Moreover, animals that did not develop symptoms within the critical time window delineated were excluded. Age- and sex-matched wild type animals of the same C57BL/6 genetic background served as young controls.

The process of physiological aging was mimicked in 92–98 weeks old C57BL/6 mice of both sexes, taking strain-identical 16–21 weeks old animals as young, mature controls.

As a model of accelerated aging, *Klotho*^−/−^ animals carrying a constitutive, hypomorphic *kl/kl* locus as described initially by Kuro-o [69] were used. Age-matched *Klotho*^+/+^ animals served as a control group. Apart from a drastically reduced survival time of 9–10 weeks [69], homozygous mutants from both sexes show a complex progeroid phenotype that overlaps with symptoms characteristic of the human aging process. These include hair greying, skin atrophy, osteoporosis, kyphosis, and arteriosclerosis. Mutants also evidence premature growth arrest coincident with impaired weight development (Supplementary Figure S1), hypogonadism, and infertility, as well as hypokinesia and defects in locomotion. Microscopic and molecular *Klotho*^−/−^ characterizations of the brain identify that this progeroid syndrome implicates, unlike other progeria models, also CNS tropisms, according to the expression of *Klotho*, e.g., in the choroid plexus [43,78]. Notably, *Klotho* expression declines with aging and therefore might contribute to the aging-related decline in structural myelin integrity [79]

Current indications suggest that this specific progeroid phenotype is, for most aspects, provoked by a systemic calcium–phosphate dyshomeostasis based on 1,25(OH)_2_D_3_, or vitamin D_3_ hypervitaminosis [80,81,82]. Molecularly, the Klotho protein operates as a co-receptor of the FGF-R1, mediating the binding of the FGF-23 bone-derived hormone, which has a role in calcium, phosphate, and vitamin D_3_ homeostasis. In accordance with recent discoveries that vitamin D_3_ deprivation obviously rescues most of the *Klotho*^−/−^ phenotypes [81], administration of a vitamin D_3_-depleted nutrition (C1017, Altromin, irradiated; <50 IU Vitamin D3/kg dietary) to counterbalance early hypercalcinosis after weaning substantially improved weight development and overall fitness and prolonged the lifespan as compared to littermates receiving standard nutrition (Supplementary Figure S1). Despite such metabolic influence on phenotypic manifestations, the implication of the neural compartment renders it suitable for progeria studies in the CNS [27].

All experimental animals were housed at the Jena University Hospital under standardized conditions for temperature, humidity, and light/dark cycles, with access to food and water *ad libitum*. The use of animals in this study was approved by the regional Animal Welfare Authorities of Thuringia (accreditation numbers: UKJ-18-004; UKJ-17-015; TWZ 01/2019). All animal-related procedures complied with current European Union regulations on the Protection of Animals Act (see section on Ethics Statements). Founder pairs of *Klotho*^+/−^ animals were kindly provided by Christoph Kaether and Heike Heuer, Fritz Lipmann Institute, Leibniz Institute on Aging, Jena, underlying informed consent by Makato Kuro-o, Division of Molecular Genetics, National Institute of Neuroscience, Tokyo, Japan.

### 4.2. Animal Anesthesia and Perfusion

Mice were successively euthanized by applying an overdose of volatile isoflurane CP^®^ (CP-Pharma, Burgdorf, Germany, Cat. 1214) until an anesthetic state of surgical tolerance and, ultimately, death was attained according to an internal standardized operating procedure (SOP) approved by local institutional animal welfare authorities. The euthanized animal was immediately transferred and fixed to a plane surgical dish. The heart was exposed and the left apex was catheterized with a knob cannula connected with a plastic tube to a perfusion mini pump. Perfusion with freshly prepared ice-cold PBS (pH 7.4) was carried out at a flow rate of 5 mL/min for 5 min to completely eliminate blood cells from the vessel system.

### 4.3. Extraction of Cortex and Spinal Cord Tissue

The mouse was detached from the perfusion set and decapitated at the cranio-cervical junction. For spinal cord extraction, the myelon was exposed under a stereomicroscope Stemi 305 trino (Zeiss, Jena, Germany, Cat. 491903-0007-000) and disconnected from surrounding structures with the aid of micro-blunted fine surgical scissors. The freshly dissected spinal cord was placed on a clean filter paper pre-wetted with ice-cold PBS in a 10 cm Petri dish (Sarstedt; Nümbrecht, Germany, Cat. 833902). Since the *hSOD1^G93A^* model’s neurodegenerative process follows an ascending caudo-rostral progression with the lumbar spinal cord being most severely affected by apoptosis, the rostral cervical and mid-thoracic regions were taken for neural cell isolation.

For extraction of cortical tissue, the brain of the decapitated mouse was exposed by removing the skull bone from both hemispheres with the help of standard pattern forceps (Fine Science Tools (FST) GmbH, Heidelberg, Germany, Cat. 91100-12). The brain was removed with the aid of a spatula (FST, Heidelberg, Germany, Cat. 10093-13) and placed on a clean filter paper pre-wetted with ice-cold PBS in a 10 cm Petri dish. The cerebellum was cut off and the two cortical hemispheres were separated using a razor blade (BAYHA^®^, Tuttlingen, Germany, Cat. 323). The hippocampus and subcortical white matter were removed with the help of fine brushes.

### 4.4. Digestion of Connective Tissue

Hibernate A-Ca (BrainBits LLC, Springfield, IL, USA, Cat. HA-CA) working solution supplemented with 0.132 M D-trehalose (Sigma-Aldrich Chemie GmbH, Darmstadt, Germany, Cat. 90208) and 310 Unit/mL of deoxyribonuclease I (Sigma-Aldrich Chemie GmbH, Darmstadt, Germany, Cat. D-5025) was prepared and used as diluent for the digestive enzymes and for the preparation of the ovomucoid (Sigma-Aldrich Chemie GmbH, Darmstadt, Germany, Cat. T9253) blocking solutions applied for the cell extraction protocol. Freshly extracted cortical bi-hemispheres (~200–220 mg) or spinal cord moieties (~60–100 mg) were transferred to a 15mL falcon tube containing 2.5 mL of collagenase type IA (Sigma-Aldrich Chemie GmbH, Darmstadt, Germany, Cat. C9891) working solution (1 mg/mL) and incubated for 30 min in an open lid-free water bath (Julabo GmbH, Seelbach, Germany, Cat. 9012327.03) at 37 °C under slight agitation. In the meantime, papain enzyme solution (Worthington Biochemical Corporation, Lakewood, NJ, USA, Cat. 3119), dissolved in 2.5 mL of HA-Ca working solution to a concentration of 2 mg/mL (papain solution I), was pre-activated by warming at 37 °C in the water bath for 30 min. After 15 min of incubation in the collagenase type IA working solution, 2.5 mL of papain solution II (1 mg/mL) was prepared in the same HA-Ca working solution and activated accordingly in the water bath at 37 °C for 30 min. The solution volumes specified here are optimized and sufficient for spinal cord or bi-hemispheric cortical preparations from one adult mouse and are generally sufficient for a tissue wet weight of ~200–220 mg. Afterwards, collagenase type IA solution was removed and substituted for digestive enzyme solutions as delineated in the following.

### 4.5. Neural Cell Isolation

A volume of 2.5 mL of pre-activated papain solution I was added to the collagenase type IA treated tissue and incubated at 37 °C for 15 min in the water bath, with gentle shaking after every 3 to 5 min. Papain solution I was collected and preserved in a separate 15 mL tube in the same water bath. Softened and enzyme-digested tissue was immersed in lower enzyme concentration papain solution II and incubated at 37 °C for another 15 min. During the incubation time, tissue disaggregation was performed by pipetting the papain solution up and down against the tissue using a pre-prepared fire-polished Pasteur pipette with a tip size of 0.75–0.80 mm. Mechanical trituration was repeated every 5 min. In total, 3 rounds of trituration comprising 7–8 strokes per round were performed. Once the tissue was disintegrated into small pieces, it was further triturated until a homogenous cell suspension was obtained. The remaining tissue pieces were allowed to settle down at the conical bottom of the 15 mL tube. The cell suspension was carefully transferred to a pre-wetted 100 µm cell strainer fixed on a 50 mL falcon tube kept on ice. Collected papain solution I was added to the extract suspension and incubated for 10 min at 37 °C, thereby continuing the trituration steps to completely digest the remaining tissue pieces. The second moiety of the cell suspension was transferred to the same 100 µm strainer used for the preceding filtering step. Then, 2.5 mL of ovomucoid blocking solution composed of 1% (*w*/*v*) ovomucoid stock solution in HA-Ca working solution at a final concentration of 0.1% (*v*/*v*) were passed through the same cell strainer to block the enzyme activity. One percent *w*/*v* ovomucoid stock solution was previously prepared and stored at −20 °C in 1 mL aliquots by dissolving 10% (*w*/*v*) BSA and 10% (*w*/*v*) ovomucoid in HA-Ca medium at a pH of 7.4. The cellular isolate was then filtered through a 40 µm cell strainer into the 50 mL tube. The cell strainer was washed with 2.5 mL of 0.1% (*v*/*v*) ovomucoid blocking solution to remove the residual cell suspension. The cell homogenate was transferred to a 15 mL falcon tube and centrifuged at 500× *g* for 5 min at room temperature (RT) in a hanging bucket centrifuge with brake and acceleration set at ~3. The supernatant was removed, and the cellular pellet was re-suspended in 1 mL of ice-cold Dulbeco’s PBS (D-PBS) (Thermo Fisher Scientific; Dreieich, Germany Cat. 14190094) containing 0.132 M D-trehalose. The cell suspension was placed on ice for ~10 min to maintain cell integrity.

### 4.6. Debris Removal Step

In order to purify the cellular isolate from debris and optimize it for flow cytometry or FACS-based applications, a debris removal step based on Miltenyis debris removal solution (Miltenyi Biotec, Gladbach, Germany, Cat. 130-109-398) was performed according to the manufacturer’s protocol, with some modifications. The cell suspension was centrifuged at 500× *g* for 5 min. Afterwards, the supernatant was completely aspirated and the cell pellet was dissolved in 1 mL of ice-cold D-PBS, with the final volume being raised up to 3 mL with D-PBS. Next, 2 mL of cold debris removal solution were added to the cell suspension and mixed by pipetting up and down (~10 times). The cell suspension was overlaid with 4 mL of cold D-PBS in the form of a continuous stream on the side of the tube, which was held in a tilted position in order to establish a gradient. The tube was centrifuged in a benchtop hanging bucket centrifuge at 3000× *g* for 10 min with full acceleration and full brake at 4 °C. The centrifugation resulted in an upper transparent phase (~4 mL), a thick white ring, a lower transparent phase (~4–5 mL), and a small white pellet at the bottom of the tube. The upper transparent phase and debris-rich ring interphase were carefully aspirated with a fire-polished glass Pasteur pipette (tip size 0.8 mm) without mixing it with the lower phase. The cell pellet was resuspended in the lower transparent phase and the final volume was made up to 7 mL with cold D-PBS and mixed by gentle inversion. The cell suspension was centrifuged at 500× *g* with brakes and acceleration reduced to ~3 at 4 °C. The cell pellet was resuspended in 1 mL of a buffer appropriate for the planned downstream applications (e.g., HBSS or D-PBS).

The cell numbers were assessed with a hemocytometer or Neubauer counting chamber. Overall viability was determined by the toluidine-derived azo dye trypan blue (Sigma-Aldrich Chemie GmbH, Darmstadt, Germany, Cat. T8154). Equal volumes (1:1) of the cell suspension and trypan blue were mixed and 10 µL were loaded to the hemocytometer. To assess cell viability within the entire cell density, the trypan blue positive, solid, rounded cells were counted as dead cells within the shiny vital population, displaying a double contour and the absence of trypan blue staining due to the preserved membrane integrity.

### 4.7. Fixation of the Isolated Neural Cells

Equal volumes of freshly prepared 4% formaldehyde were added to the cell suspension to achieve a final concentration of 2% formaldehyde. Cells were incubated for 20 min at RT and centrifuged at 500× *g* for 5 min. The supernatant was removed and the cells were washed twice with PBS. Fixed cells can be preserved in PBS at 4 °C and processed further, e.g., for immunostaining, within 48 h. Longer latencies might bear the risk of increased autofluorescence.

### 4.8. Quenching of Unspecific Fluorescence Signals

The autofluorescence quenching step was optionally performed after the debris removal step, given that downstream assay reagents excluded permeabilizing agents. In case detergents were involved, the quenching step was immediately performed after immunostaining, to avoid the risk of purging the quencher from the treated cells. This step is particularly relevant in the context of senescence studies, as lipofuscin continues to occupy an integral role in the phenomapping of senescence [34]. In the aging CNS, its specificity is supposed to be superior to that of SA-β-gal, given that, in this environment, lysosomal β-galactosidase might dominate over or be indistinguishable from senescence-associated SA-β-galactosidase signals [66,67].

Cells were resuspended in 50 µL of the 0.0625× lipofuscin autofluorescence quencher working solution (PromoCell, GmbH, Heidelberg, Germany, Cat. PK-CA707-23007) in 70% ethanol for 1 min at RT. To define the optimal concentrations, a serial dilution of the quencher stock solution was generated in 70% ethanol (0.0625×, 0.125×, 0.25×, and 0.5× up to 1× from the 20× stock solution). The cells were then centrifuged at 700× *g* for 5 min, the supernatant was removed, and the cells were washed in 200 µL of 70% ethanol. The suspension was again centrifuged at 700× *g* for 5 min, the supernatant-free cells were washed with 200 µL of D-PBS, and the final pellet was resuspended in 200 µL D-PBS.

### 4.9. Immunostaining for Flow Cytometry-Based Separation of Neural Cell Entities

The cells were resuspended in 1 mL of D-PBS. This volume was equally divided into 1.5 mL reaction tubes according to the cell numbers calculated, aiming to achieve a preferred cell number of ~5 × 10^5^–7 × 10^5^ cells per 1.5 mL tube. Cells were centrifuged at 500× *g* to remove the supernatant. Non-specific antigen sites were blocked by the addition of 250 µL of blocking solution composed of 10% normal donkey sera (NDS; GeneTex; Eching, Germany, Cat. GTX 7324 5-500) in 3% bovine serum albumin (BSA; Serva Electrophoresis GmbH; Heidelberg, Germany, Cat. 11930.03) solution in PBS to each of the cell suspension aliquots and incubated at RT for 3 h with continuous rotation. Fixed cells can be blocked for 3–4 h at RT; alternatively, the blocking step can be extended to an overnight period at 4 °C without affecting the staining properties. The cells were centrifuged at 500× *g* and the supernatant was removed. Afterwards, 250 µL of primary antibody solution in antibody diluent or antibody-free diluent (10% NDS in 3% BSA solution in PBS with 0.3% of permeabilizing agent Triton X-100 (Merck KGaA, Darmstadt, Germany, Cat. 1086031000)) were added for the assessment of specific markers, as detailed in Table 2. Preparations omitting primary antibodies were carried out to define the autofluorescence threshold parameters and background intensities arising from secondary antibody controls. Anti-CD68, which lacks expression in CNS-intrinsic cell populations, was used to depict the specificity of primary antibodies. Data described in Table 2 summarize the antibody concentrations optimized by titration for the conditions delineated in this study. After antibody application, cells were incubated under constant low rotation on a loopster (IKA, Staufen, Germany, Cat. 100314088) at 4 °C overnight. Afterwards, the cells were washed twice with PBS and the corresponding secondary antibody solution was added (Table 2) and incubated at RT for 30 min under slow but constant rotation. The cells were washed twice with PBS and resuspended in 300 µL of D-PBS. Stained cells can be preserved at 4 °C for up to 24 h. Expanded incubation times might increase autofluorescence.

### 4.10. Flow Cytometry-Based Assessment of Different Neural Cell Proportions

**Figure 12:**
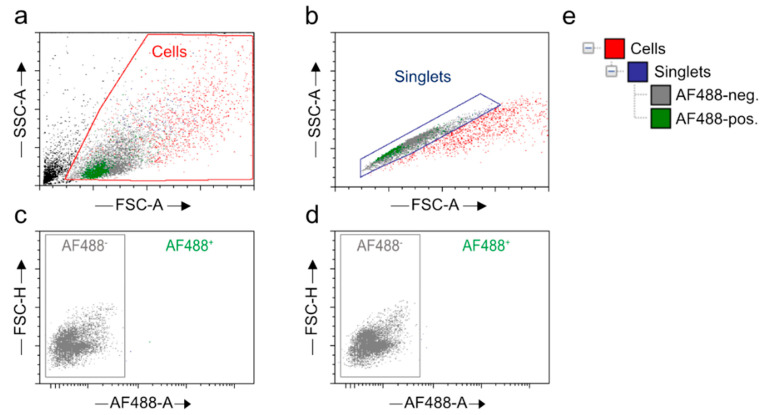
Gating strategies for neural cell selection from the CNS isolate. Scatter plots exemplifying flow cytometric cell events from a young wild type C57BL/6 cortex. (**a**–**d**) Representative dot plots generated on the FACSAria^TM^ Fusion device and visualized with De Novo FCS Express version 5 Plus Flow Cytometry software: (**a**) Black moiety, representing debris and apoptotic cells, is excluded and vital cells are gated as ‘Cells’ on the basis of size (FSC-A) and granularity (SSC-A) (displayed in red). (**b**) Doublets are excluded. Singlets are selected on the basis of size and identified by their stringent, linear relation of FSC-A and FSC-H parameters represented by x- and y-axes. (**c**,**d**) The AF488^−^ population, discriminated by (**c**) in absence of any antibody staining and (**d**) under control application of an appropriate secondary antibody while omitting a primary marker antibody, is excluded and thus distinguishes autofluorescence from specific signal events. (**e**) Gate view for sequentially identified neural subpopulations. Red, vital population; blue, singlets within vital moiety; grey, autofluorescence; green, AF488^+^ target population encompassing vital singlets. FSC-A, forward scatter-area; SSC-A, side scatter-area, AF488-A, Alexa Fluor 488-area, FSC-H, forward scatter-height.

The cells were passed through a 30 µm cell strainer (Miltenyi Biotec, Gladbach, Germany, Cat. 130-041-407) into 5 mL round-bottom polypropylene tubes (Fisher Scientific, Schwerte, Germany, Cat. 352008). Flow cytometry was performed using an appropriate nozzle size and laser and voltage power. All adjusted parameters and approved settings were kept identical for the subsequent runs, except for tolerating minor inter-run (AF488) adjustments. The target cell types were selected using appropriate gating strategies as described (Figure 12). The vital cell proportions were selected, and debris was excluded by plotting a dot plot between the SSC area (SSC-A) representing granularity and FSC area (FSC-A), which is proportional to the size properties of the cells (Figure 12a). Doublets were stringently excluded by generating a dot plot between FSC-height (FSC-H) and FSC-A (Figure 12b). To define the specificity of the target populations, the gates were set for autofluorescence using tissue- and age-specific unstained and secondary antibody stained (omitting primary antibody) controls (Figure 12c,d).

Neuronal populations from the brain or spinal cord isolates were selected by generating a dot plot between AF488-βIII-tubulin/Tuj1-A and FSC-H (Figure 4a,f and Figure 6a,f). Similarly, a dot plot was created between AF488-S100β-A and FSC-H for the selection of astrocytes (Figure 4b,g and Figure 6b,g). A separate dot plot was conceived between AF488-Iba1-A and FSC-H to distinguish the microglial population (Figure 4c,h and Figure 6c,h). Likewise, oligodendrocytes were selected from CAII-stained samples by plotting a dot plot between AF488-CAII-A and FSC-H (Figure 4d,i and Figure 6d,i). An additional dot plot was generated between AF488-CD68-A and FSC-H to show primary antibody specificity (Figure 4e,j and Figure 6e,j). For each sample, 1 × 10^4^ events were recorded. Refined data analyses, including the assessment of cell type-specific percentages, were realized by the same plotting strategy with the aid of the De Novo FCS Express version 5 Plus Flow Cytometry software (De Novo Software, Pasadena, CA, USA) (Figure 4 and Figure 6) [83].

### 4.11. Quantification and Statistical Analysis

Data were analyzed and quantified by means of the De Novo FCS Express version 5 Plus Flow Cytometry software (De Novo Software, Pasadena, CA, USA) [83]. All values were displayed as means ± standard error of the mean (SEM). Statistics were calculated using GraphPad Prism version 8.4.2 (GraphPad Software, San Diego, CA, USA) [84]. In detail, for comparisons of two groups, an unpaired two-tailed Student’s t-test was executed assuming a normal distribution and equal variance. For multiple comparisons of normally distributed data, one- or two-way Analysis of Variance (ANOVA) were exerted. Corrections for multiple comparisons were based on Holm-Šidák, Tukey, or Šidák post hoc tests. A p-value of < 0.05 was considered statistically significant. The details on the statistical tests executed and the underlying numbers are specified in the respective figure legends.

## Figures and Tables

**Table 1:** Clinical scoring parameters for standardized assessment of ALS-like motor symptoms in mice.

Clinical Score	Clinical Manifestation
**0**	Absence of symptoms defined by the ability of the mouse to fully extend its hind limbs away from the body midline at least for 2 s during the tail suspension test (repeated 2–3 times).
**1**	The mouse shows a collapse of its hind limbs extension away from the body midline or a trembling of the hind limbs during tail suspension, indicating a beginning of hind limb paresis.
**2**	The mouse is dragging along any part of the feet or curling the toes during walking.
**3**	Signs of rigid paresis appear along with the feet not being used for forward motion.
**4**	The mouse is not able to right itself within 30 s from either side. Therewith, the human endpoint is reached.

**Table 2:** Antibodies used for immunofluorescence linked to FACS.

1st Antibody	2nd Antibody	Final Dilution 1st Antibody	Final Dilution 2nd Antibody	Volume (per Sample)
**Rabbit anti-βIII-tubulin RRID:** AB_262133	Alexa Fluor^®^ 488-conjugated donkey anti-rabbit IgG (H + L) **RRID:** AB_2535792	1:3000	1:2000	250 µL
**Rabbit anti-CA-II RRID:** AB_2065996		1:250	1:2000	250 µL
**Rabbit anti-Iba-1 RRID:** AB_839504		1:500	1:2000	250 µL
**Rabbit anti-S100βRRID:** AB_2620024		1:5000	1:2000	250 µL
**Rat anti-CD68RRID:** AB_324217	Alexa Fluor^®^ 488-conjugated goat anti-rat IgG (H + L) **RRID:** AB_2534074	1:100	1:2000	250 µL

## Data Availability

Not applicable.

## Supplementary Materials

The following supporting information can be downloaded at: www.mdpi.com/article/10.3390/ijms23063000/s1:
