# Supplementary material for: Optimized Protocol for Proportionate CNS Cell Retrieval as a Versatile Platform for Cellular and Molecular Phenomapping in Aging and Neurodegeneration
Source: Int J Mol Sci. 2022 Mar 10;23(6):3000. doi: 10.3390/ijms23063000 (PMC8950438; doi:10.3390/ijms23063000)
Supplement: Supplementary file 1 [file ijms-23-03000-s001.zip › ijms-1607199-supplementary.pdf]

Supplementary Table S1. Absolute cellular yield from young and aged murine cortex without debris removal step. n, number of isolates considered.

Absolute cellular yield from young and aged cortical isolates (average ± SEM)			
**Group**	**Age (weeks)**	**Absolute cellular yield**	**n**
**Young**	20-22	~$7.2 \times 10^6 \pm 6.3 \times 10^5$	3
**Aged**	94-97	~$6.2 \times 10^6 \pm 8.7 \times 10^5$	3

Supplementary Table S2. Cellular yield of individual neural subpopulations isolated from cortex, expressed in absolute numbers. n, number of isolates considered.

Absolute numbers of different neural cell types in cortical isolates (average ± SEM)					
**Group**	**Parameter**	**Neurons**	**Astrocytes**	**Microglia**	**Oligodendrocytes**
Wild type	absolute cell number	~$1.6 \times 10^6 \pm 1.8 \times 10^5$	~$6.3 \times 10^5 \pm 6.2 \times 10^4$	~$1.3 \times 10^6 \pm 1.1 \times 10^5$	~$7.2 \times 10^5 \pm 1.5 \times 10^5$
	n	6	6	6	3
hSOD1^{G93A}	absolute cell number	~$1.1 \times 10^6 \pm 1.4 \times 10^5$	~$4.3 \times 10^5 \pm 5.7 \times 10^4$	~$6.5 \times 10^5 \pm 9.4 \times 10^4$	~$5.0 \times 10^5 \pm 7.7 \times 10^4$
	n	6	6	6	3

Supplementary Table S3. Cellular yield of individual neural subpopulations isolated from spinal cord, expressed in absolute numbers. n, number of isolates considered.

Absolute numbers of different neural cell types in spinal isolates (average ± SEM)					
**Group**	**Parameter**	**Neurons**	**Astrocytes**	**Microglia**	**Oligodendrocytes**
Wild type	absolute cell number	~$4.9 \times 10^5 \pm 1.3 \times 10^5$	~$2.6 \times 10^5 \pm 2.9 \times 10^4$	~$1.5 \times 10^5 \pm 2.8 \times 10^4$	~$7.1 \times 10^4 \pm 6.7 \times 10^3$
	n	4	4	4	4
hSOD1^{G93A}	absolute cell number	~$3.3 \times 10^5 \pm 3.9 \times 10^4$	~$1.3 \times 10^5 \pm 1.7 \times 10^4$	~$2.3 \times 10^5 \pm 4.7 \times 10^4$	~$7.1 \times 10^5 \pm 1.4 \times 10^4$
	n	5	6	5	6

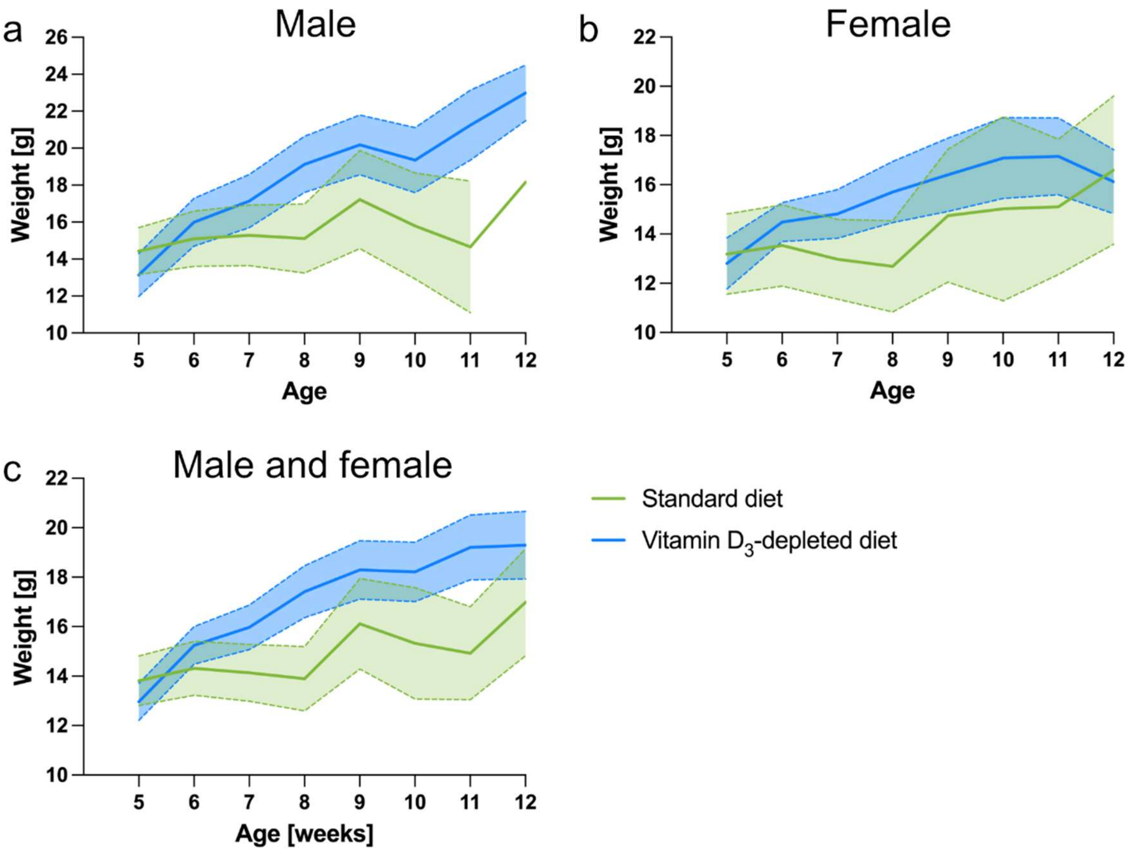

Supplementary Figure S1. Weight curve of hypomorphic *Klotho*$^{-/-}$ animals supplied with standard versus vitamin D_3-depleted diet. Post-weaning supply with vitamin D_3-depleted nutrition stabilized body weight of *Klotho*$^{-/-}$ mice as compared to Klotho$^{+/+}$ control mice fed with standard diet. The curves represent animals of both sexes in balanced proportions (n = 14 per group). At the end of the observation period, only 3 animals had survived in the standard diet group, while 13 mice remained in the treatment group.
